# Supplementary material for: Nanocrown electrodes for parallel and robust intracellular recording of cardiomyocytes
Source: Nat Commun. 2022 Apr 26;13:2253. doi: 10.1038/s41467-022-29726-2 (PMC9042818; doi:10.1038/s41467-022-29726-2)
Supplement: Supplementary file 2 — Reporting Summary [file 41467_2022_29726_MOESM2_ESM.pdf]

nature portfolio

Corresponding author(s): Bianxiao Cui

Last updated by author(s): Mar 6, 2022

Reporting Summary

Nature Portfolio wishes to improve the reproducibility of the work that we publish. This form provides structure for consistency and transparency in reporting. For further information on Nature Portfolio policies, see our Editorial Policies and the Editorial Policy Checklist.

Statistics

For all statistical analyses, confirm that the following items are present in the figure legend, table legend, main text, or Methods section.

n/a	Confirmed	
☐	☒	The exact sample size (*n*) for each experimental group/condition, given as a discrete number and unit of measurement
☐	☒	A statement on whether measurements were taken from distinct samples or whether the same sample was measured repeatedly
☒	☐	The statistical test(s) used AND whether they are one- or two-sided<br>*Only common tests should be described solely by name; describe more complex techniques in the Methods section.*
☒	☐	A description of all covariates tested
☒	☐	A description of any assumptions or corrections, such as tests of normality and adjustment for multiple comparisons
☐	☒	A full description of the statistical parameters including central tendency (e.g. means) or other basic estimates (e.g. regression coefficient) AND variation (e.g. standard deviation) or associated estimates of uncertainty (e.g. confidence intervals)
☒	☐	For null hypothesis testing, the test statistic (e.g. *F*, *t*, *r*) with confidence intervals, effect sizes, degrees of freedom and *P* value noted<br>*Give P values as exact values whenever suitable.*
☒	☐	For Bayesian analysis, information on the choice of priors and Markov chain Monte Carlo settings
☒	☐	For hierarchical and complex designs, identification of the appropriate level for tests and full reporting of outcomes
☒	☐	Estimates of effect sizes (e.g. Cohen's *d*, Pearson's *r*), indicating how they were calculated

Our web collection on statistics for biologists contains articles on many of the points above.

Software and code

Policy information about availability of computer code

Data collection	MATLAB (Version R2018a, MathWorks)<br>MC_Rack (version 4.6.2, Multi Channel Systems)<br>MC_DataTool (version 2.6.15 MultiChannelSystems)<br>pClamp (Version 11, Molecular Devices
Data analysis	MATLAB (Version R2018a, MathWorks)<br>Prism (Version 7.04, GraphPad software)<br>ImageJ (Version 1.52a, National Institute of Health)

For manuscripts utilizing custom algorithms or software that are central to the research but not yet described in published literature, software must be made available to editors and reviewers. We strongly encourage code deposition in a community repository (e.g. GitHub). See the Nature Portfolio guidelines for submitting code & software for further information.

Data

Policy information about availability of data

All manuscripts must include a data availability statement. This statement should provide the following information, where applicable:

- Accession codes, unique identifiers, or web links for publicly available datasets
- A description of any restrictions on data availability
- For clinical datasets or third party data, please ensure that the statement adheres to our policy

All the raw data included in the figures are now publicly available at https://drive.google.com/drive/folders/1VNJkzDY0OjxNbCOzYozcPfSIjT2LbWo1?usp=sharing. We are currently exploring the potential of commercializing the analysis software. The analysis code will be available upon request from researchers in not-for-

profit organizations.

Field-specific reporting

Please select the one below that is the best fit for your research. If you are not sure, read the appropriate sections before making your selection.

☒ Life sciences ☐ Behavioural & social sciences ☐ Ecological, evolutionary & environmental sciences

For a reference copy of the document with all sections, see nature.com/documents/nr-reporting-summary-flat.pdf

Life sciences study design

All studies must disclose on these points even when the disclosure is negative.

Sample size	We did not perform calculations to determine the sample size. All experiments were repeated for at least three times, sometimes with N>300 independent measurements. The success rate is >99%. The technology is highly robust and repeatable among different samples.
Data exclusions	No data exclusions.
Replication	Experiments were repeated at least three times and each experiments usually consists of 10 or more independent measurements. For statistics, 80-300 measurements were pooled together. As mentioned above, the success rate is >99%.
Randomization	Not applicable. This is a technology development work. The success rate is defined as the ability to record intracellular potential upon a single electroporation pulse for any cell that shows extracellular signals before electroporation. The final success rate is calculated for >1000 indepenpendent measurements. All data points are included. Randomization is not applicable.
Blinding	Not applicable. This is a technology development work. We compare extracellular signal and intracellular signal from the same cell. Blinding is not applicable.

Reporting for specific materials, systems and methods

We require information from authors about some types of materials, experimental systems and methods used in many studies. Here, indicate whether each material, system or method listed is relevant to your study. If you are not sure if a list item applies to your research, read the appropriate section before selecting a response.

Materials & experimental systems

n/a	Involved in the study	
☐	☒	Antibodies
☐	☒	Eukaryotic cell lines
☒	☐	Palaeontology and archaeology
☒	☐	Animals and other organisms
☒	☐	Human research participants
☒	☐	Clinical data
☒	☐	Dual use research of concern

Methods

n/a	Involved in the study	
☒	☐	ChIP-seq
☒	☐	Flow cytometry
☒	☐	MRI-based neuroimaging

Antibodies

Antibodies used	Anti-integrin beta1 antibody: anti-Itgb1 is from Sigma-Aldrich, Cat#: MAB2079Z, Lot#:2159648.<br>Secondary antibody Goat anti-Mouse IgG (H+L) Cross-Adsorbed Secondary Antibody, Alexa Fluor 488: Thermo Fisher Scientific, Cat#: A11001
Validation	The anti-integrin beta1 antibody (MAB2079Z) has been widely used by many researchers. The validation is available through supplier's website. We independently validated this antibody in our lab by the characteristic staining of focal adhesion patches.

Eukaryotic cell lines

Policy information about cell lines

Cell line source(s)	The iPSC-CMs we used were differentiated from iPSC line SCVI-273 obtained from Stanford Cardiovascular Institute iPSC Biobank. It is an iPSC line derived from healthy individuals.<br>U2OS human bone osteosarcoma epithelial cells (ATCC® HTB-96™)
Authentication	iPSC-CMs are authenticated by observing its characteristic mechanical contraction (iPSC is directly from the Stanford

	Cardiovascular Institute iPSC biobank). U2OS cells were recently purchased from ATCC and the passage were carefully documented.
Mycoplasma contamination	U2OS cells were tested and negative of mycoplasma. iPSCs were not tested in the lab. The iPSCs were used for differentiation and not further passaged in our lab.
Commonly misidentified lines (See ICLAC register)	No misidentified lines were used in this study.
